# *ALOX5* exhibits anti-tumor and drug-sensitizing effects in *MLL*-rearranged leukemia

**DOI:** 10.1038/s41598-017-01913-y

**Published:** 2017-05-12

**Authors:** Yungui Wang, Jennifer R. Skibbe, Chao Hu, Lei Dong, Kyle Ferchen, Rui Su, Chenying Li, Hao Huang, Hengyou Weng, Huilin Huang, Xi Qin, Jie Jin, Jianjun Chen, Xi Jiang

**Affiliations:** 1 Department of Hematology, the First Affiliated Hospital Zhejiang University College of Medicine, Hangzhou, Zhejiang 310003 China; 2 Department of Cancer Biology, University of Cincinnati, Cincinnati, OH 45219 USA; 3 Section of Hematology/Oncology, Department of Medicine, University of Chicago, Chicago, IL 60637 USA; 4 Department of Obstetrics and Gynecology, Feinberg School of Medicine, Northwestern University, Chicago, IL 60611 USA

## Abstract

*MLL*-rearranged acute myeloid leukemia (AML) remains a fatal disease with a high rate of relapse and therapeutic failure due to chemotherapy resistance. In analysis of our Affymetrix microarray profiling and chromatin immunoprecipitation (ChIP) assays, we found that *ALOX5* is especially down-regulated in *MLL*-rearranged AML, *via* transcription repression mediated by Polycomb repressive complex 2 (PRC2). Colony forming/replating and bone marrow transplantation (BMT) assays showed that Alox5 exhibited a moderate anti-tumor effect both *in vitro* and *in vivo*. Strikingly, leukemic cells with *Alox5* overexpression showed a significantly higher sensitivity to the standard chemotherapeutic agents, i.e., doxorubicin (DOX) and cytarabine (Ara-C). The drug-sensitizing role of *Alox5* was further confirmed in human and murine *MLL*-rearranged AML cell models *in vitro*, as well as in the *in vivo MLL*-rearranged AML BMT model coupled with treatment of “5 + 3” (i.e. DOX plus Ara-C) regimen. Stat and K-Ras signaling pathways were negatively correlated with *Alox5* overexpression in *MLL-AF9*-leukemic blast cells; inhibition of the above signaling pathways mimicked the drug-sensitizing effect of *ALOX5* in AML cells. Collectively, our work shows that *ALOX5* plays a moderate anti-tumor role and functions as a drug sensitizer, with a therapeutic potential, in *MLL*-rearranged AML.

## Introduction

Acute myeloid leukemia (AML), the most common type of acute leukemia attacking adults, is a heterogeneous disease characterized by the malignant expansion of dysfunctional myeloid progenitors and the suppression of normal hematopoiesis^1^. The *mixed lineage leukemia* (*MLL*) gene on chromosome 11q23 is the frequent target of chromosomal translocations and rearrangements in infant and adult leukemias. AML bearing *MLL* fusion often associates with poor prognosis and is resistant to most of the current clinical chemotherapies^2–4^. Though immense efforts have been exerted in developing novel therapeutic strategies in the past decades, the standard chemotherapy with intensive administration of chemotherapeutic drugs, e.g. the “7 + 3” regimen (i.e. combined treatment of anthracycline (daunorubicin or doxorubicin) and cytarabine (Ara-C)), remains the main therapeutic approach for treating AML, including *MLL*-rearranged AML^5–8^. Resistance to the standard chemotherapy remains the major cause of relapse and therapeutic failure^7, 9^. Despite intensive chemotherapy, the overall survival of AML patients is still less than 30% for adults and 60% for children, and most of the patients with *MLL*-rearranged AML fail to survive longer than 5 years^4, 9–11^. Therefore, it is crucial to understand the mechanisms underlying the pathogenesis and chemotherapy resistance of AML, such as *MLL*-rearranged AML, and identify potential new therapeutic targets that can suppress AML or improve the response to the currently existing chemotherapies.

The mechanism of AML drug response remains obscure. A number of key factors including transcriptional factors, oncogenes and tumor suppressors, as well as cell metabolism regulators, have been identified to play important roles in determining drug sensitivity or resistance^7, 9^. Among these factors, the JAK/STAT signaling is known to be closely associated with myeloid malignancies. Activation of JAK/STAT pathway is a hallmark of the myeloproliferative neoplasms, and is found in many AML cases^12, 13^. JAK/STAT pathway inhibitors, e.g. pacritinib and ruxolitinib, have already been studied in pre-clinical and clinical trials^14, 15^. The proto-oncogene K-RAS is another factor involved in both leukemogenesis and therapeutic response^16^. *K-RAS* mutations have been reported in many AML cases and are associated with prognosis and chemotherapy resistance^17, 18^. It holds great potential to cure AML by either targeting these factors directly or targeting a common upstream regulator that controls all these factors in AML.

The arachidonate 5-lipoxygenase gene (*ALOX5*) encodes a non-heme iron-containing enzyme of the lipoxygenase family. It catalyzes the production of leukotrienes (LTs) and reactive oxygen species (ROS) from arachidonic acid^19, 20^. ALOX5 is known to be involved in various physiological and pathological processes, including oxidative stress response, inflammation and cancer^21–25^. It was reported previously that loss of *Alox5* impairs the function of leukemic stem cells (LSCs) in *BCR-ABL*-induced chronic myelogenous leukemia (CML)^26^. To our surprise, our gene profiling reveals that *ALOX5* expression is particularly down-regulated in *MLL*-rearranged AML. Both *in vitro* and *in vivo* studies were carried out to investigate the effects and underlying mechanism of *ALOX5* in AML pathogenesis and drug response.

## Results

### *ALOX5* is especially down-regulated in *MLL*-rearranged AML

**Figure 1:**
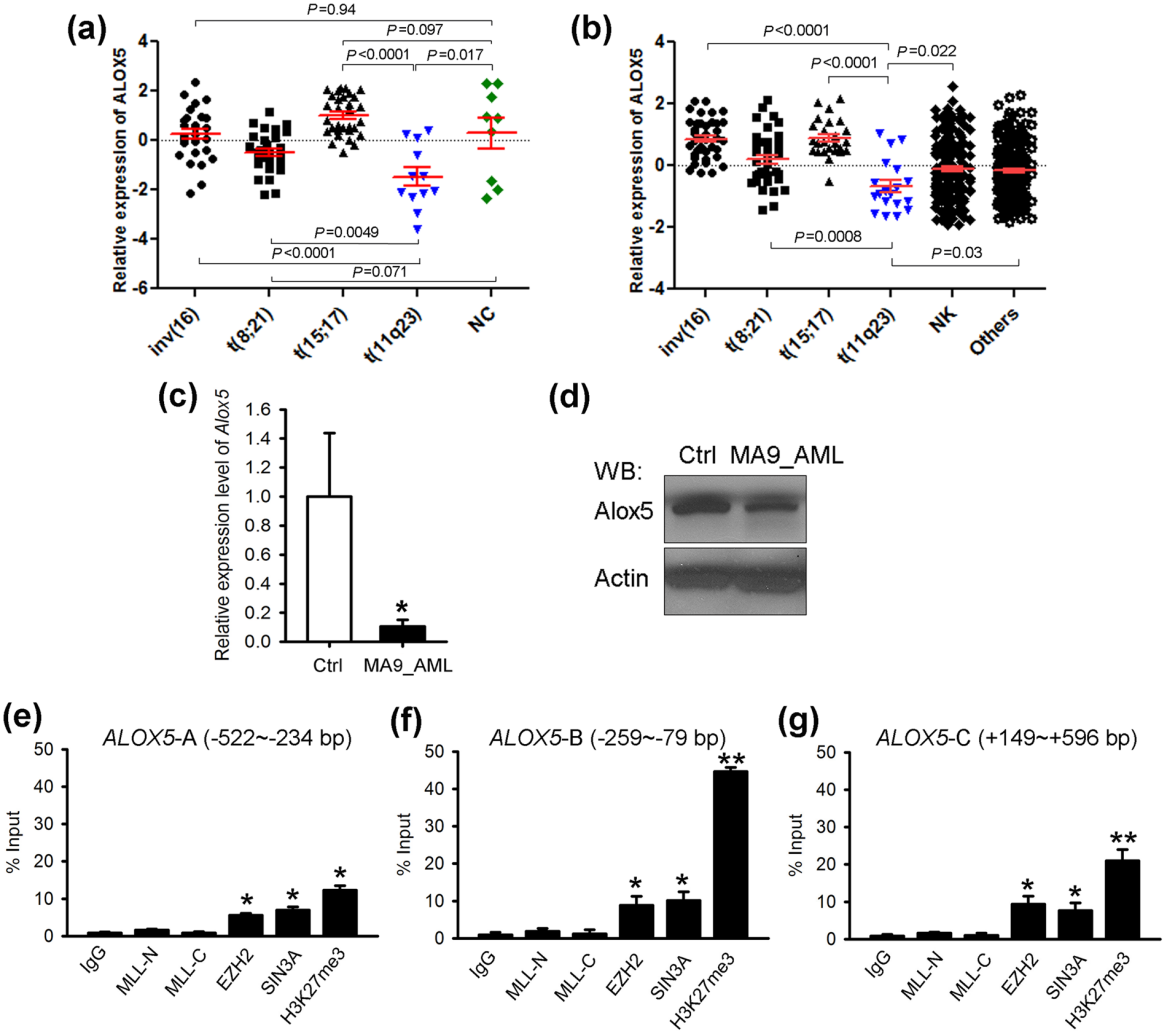
Expression of *ALOX5* is significantly down-regulated in *MLL*-rearranged AML. (**a**) Comparison of *ALOX5* expression between t(11q23)/*MLL*-rearranged AML and AML cases with inv(16), t(8;21) or t(15;17), or normal controls (NC) in our in-house AML dataset^27–29^. (**b**) Comparison of *ALOX5* expression between t(11q23) AML and other subtypes of AML cases in GSE14468^30^. The expression values were log2-transformed and mean centered. The *P* values were detected by *t*-test. (**c**,**d**) Downregulation of *Alox5* in *MLL-AF9*-AML mouse BM mononuclear cells, as compared with normal controls. Levels of Alox5 were detected through qPCR (**c**) and Western blotting (**d**). (**e**–**g**) *EZH2* and *SIN3A* bind the promoter of *ALOX5*. Enrichment of MLL-N terminal (for both wild-type MLL and MLL fusions), MLL-C terminal (for wild-type MLL), EZH2, SIN3A, H3K27me3 or IgG at the *ALOX5* promoter region was shown. Relative to transcription start site of *ALOX5*, locations of the three PCR amplicon sites are: *ALOX5*-A (**e**): −522~−234 bp; *ALOX5*-B (**f**): −259~−79 bp; *ALOX5*-C (**g**): +149~+596 bp^32^. *P < 0.05; **P < 0.01, two-tailed *t*-test.

In analysis of our Affymetrix microarray-based gene expression profiling of 100 human AML samples carrying *MLL*-rearrangements/t(11q23) (n = 12), inv(16) (n = 27), t(8;21) (n = 30) or t(15;17) (n = 31), along with 9 normal bone marrow (BM) control (3 each for CD34^+^ hematopoietic stem/ progenitor cell, CD33^+^ myeloid progenitor cell, and mononuclear cell (MNC)) samples^27–29^, we found that *ALOX5* was expressed at a significantly lower level in *MLL*-rearranged AML, but not in other AML subtypes, relative to the normal controls (Fig. 1a). Notably, the expression level of *ALOX5* in *MLL*-rearranged AML is also significantly lower than that in other AML subtypes (Fig. 1a); such pattern was also observed in an independent large-scale AML dataset (i.e., GSE14468^30, 31^) (Fig. 1b). The downregulation of Alox5 in *MLL*-rearranged AML was verified through both qPCR (Fig. 1c) and Western blotting (Fig. 1d) in *MLL-AF9* leukemic blast cells isolated from the relevant leukemic mice, as compared with normal controls.

To understand the mechanisms of the repression of *ALOX5* in *MLL*-rearranged AML, we conducted chromatin immunoprecipitation (ChIP) assays. We found no significant enrichment of MLL fusion proteins at the promoter region^32^ of the *ALOX5* locus (Fig. 1e–g). It is known that gene silencing mediated by the Polycomb repressive complex 2 (PRC2) and cofactors, e.g. EZH2 and SIN3A, is essential for *MLL*-rearranged AML^33^. Here we show a significant enrichment of EZH2 and SIN3A at the *ALOX5* promoter region. Histone H3 lysine 27 tri-methylation (i.e., H3K27me3), a marker for repressive transcription associated with EZH2^34, 35^, also exhibited high enrichement at these genomic locus (Fig. 1e–g). Therefore, it is highly likely that the PRC2 complex mediates the transcriptional repression of *ALOX5* in *MLL*-rearranged leukemia.

### Anti-tumor effect of *Alox5* in *MLL*-rearranged AML

**Figure 2:**
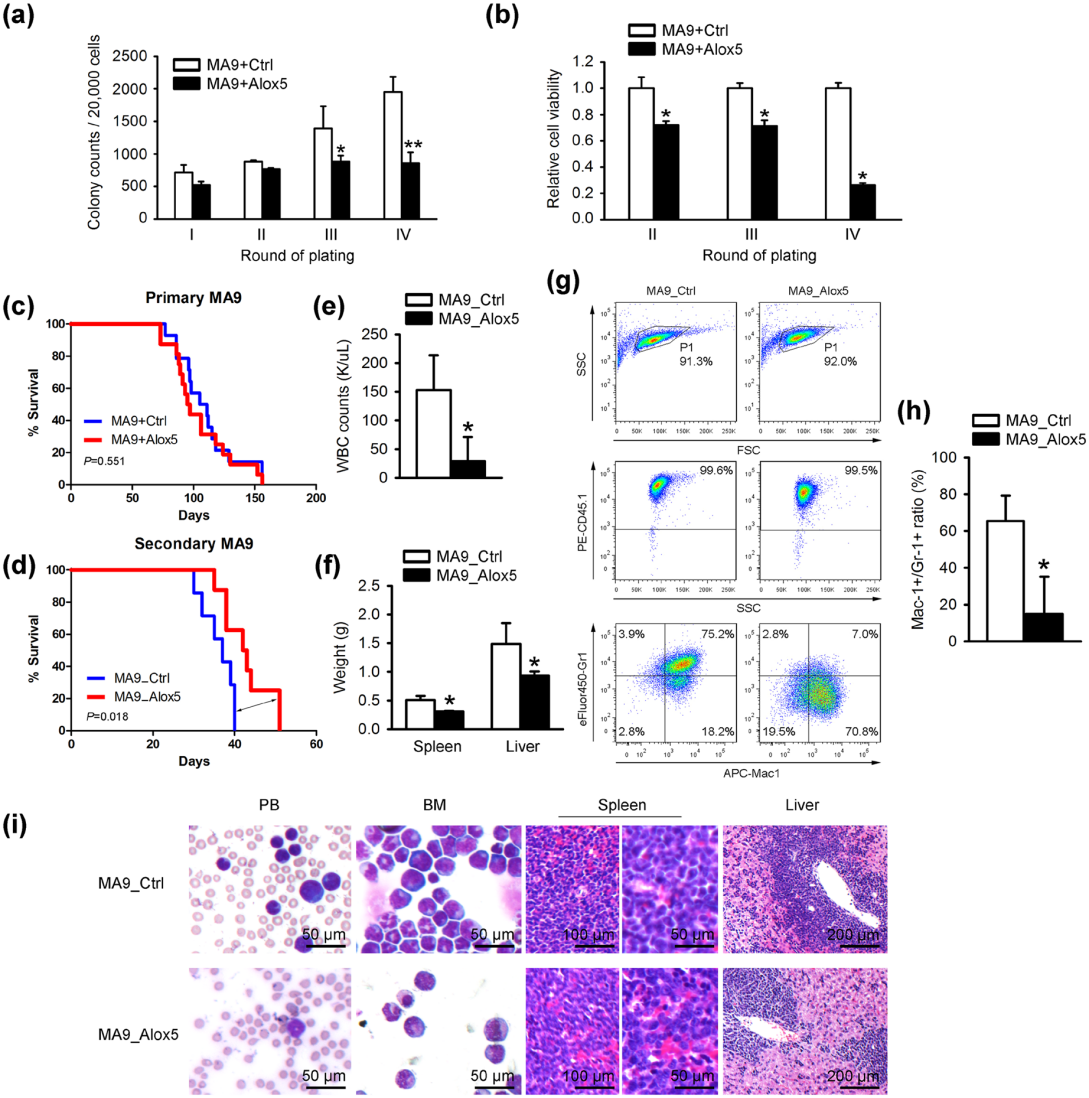
Anti-tumor effect of *Alox5* in *MLL-AF9*-AML. (**a**) Inhibitory effect of *Alox5* on *MLL-AF9*-induced colony forming/immortalization of normal mouse bone marrow progenitor cells. Normal mouse bone marrow progenitor cells were retrovirally transduced with MSCVneo-*MLL-AF9* + MSCV-PIG (MA9 + Ctrl) or MSCVneo-*MLL-AF9* + MSCV-PIG-*Alox5* (MA9 + Alox5). Colony forming/replating assays (CFAs) were conducted for four passages. 20,000 cells were plated in each dish. (**b**) *Alox5* overexpression represses cell viability of *MLL-AF9*-transduced mouse bone marrow progenitor cells. After the first passage, 10,000 of the above transduced cells were seeded in liquid culture medium. MTT assays were conducted 72 hours after seeding. (**c**) Effects of *Alox5* on *MLL-AF9*-induced primary leukemia *in vivo*. Normal mouse bone marrow progenitor cells (CD45.1) were retrovirally co-transduced with MSCVneo-*MLL-AF9* + MSCV-PIG (MA9 + Ctrl) or MSCVneo-*MLL-AF9* + MSCV-PIG-*Alox5* (MA9 + Alox5). 0.2 × 10^5^ of the transduced cells were used as donor cells and transplanted into each primary recipient mouse (CD45.2). MA9 + Ctrl: n = 14; MA9 + Alox5: n = 16. (**d**) Inhibitory effects of *Alox5* on the maintenance of *MLL-AF9*-induced AML in secondary BMT recipient mice. The secondary BMT recipients were transplanted with BM blast cells from the primary *MLL-AF9* AML mice retrovirally transduced with MSCV-PIG (MA9-AML_Ctrl; n = 7) or MSCV-PIG-*Alox5* (MA9-AML_Alox5; n = 8). Kaplan-Meier curves are shown. The *P* values were determined by log-rank test. (**e**) Total white blood cell (WBC) counts of the secondary BMT recipients were determined by Hemavet 950 at the mice’ end points. n = 5 for each group. (**f**) Weights of spleen and liver of each secondary BMT recipient at its end point are shown. n = 5 for each group. (**g**) Flow cytometry analyses of BM cells of secondary BMT recipients. The “blast” population was gated by FSC/SSC (framed out; upper panel) and the proportions of CD45.1^+^ (middle panel), Gr1^+^ and/or Mac1^+^ cells were analyzed (lower panel). (**h**) Statistical analysis of the Gr1^+^Mac1^+^ population of secondary BMT recipients. n = 5 for each group. *P < 0.05; **P < 0.01, two-tailed *t*-test. (**i**) Wright-Giemsa staining of mouse peripheral blood (PB) and BM, or hematoxylin and eosin (H&E) staining of mouse spleen and liver of secondary BMT recipients.

To assess the pathological role of *Alox5* in *MLL*-rearranged AML, we cloned *Alox5* CDS into MSCV-PIG retroviral vector, and then co-transduced MSCVneo-*MLL-AF9* (MA9) with MSCV-PIG-*Alox5* (Alox5) or MSCV-PIG (Ctrl) into mouse BM progenitor cells for *in vitro* colony-forming/replating assays. We showed that along with the increased number of passages, *Alox5* overexpression showed a more significant degree of repression on *MLL-AF9*-induced colony forming (Fig. 2a). We also analyzed cell viability of those retrovirus transduced progenitor cells and showed that forced expression of *Alox5* significantly suppressed cell viability (Fig. 2b). Knockdown of *ALOX5* with siRNA did not show significant alterations in the viability of MONOMAC-6/t(9;11) cells (Supplementary Figs 1a–c and 3a,b).

In order to determine the *in vivo* effect of *Alox5* in leukemogenesis, we performed primary BM transplantation (BMT) assay first and found that forced expression of *Alox5* showed no significant influence on overall survival (medium overall survival: 108 days for the MA9 + Ctrl group *vs*. 96 days for MA9 + Alox5; *P* = 0.556, log-rank test) (Fig. 2c). We then isolated the leukemic blast cells from primary *MLL-AF9*-leukemic mice and transduced the cells with *Alox5* or control retrovirus, and performed secondary BMT assays. Notably, *Alox5* overexpression significantly delayed the progression of *MLL-AF9* AML in secondary BMT recipients (median overall survival, 42.5 days (MA9_Alox5) *vs*. 37 days (MA9_Ctrl); *P* = 0.018, log-rank test) (Fig. 2d). While all the leukemic mice died from AML, overexpression of *Alox5* resulted in a significant decrease in peripheral white blood cell count (Fig. 2e) and spleen size (Fig. 2f). Moreover, through flow cytometry analysis, we found that although the *Alox5* overexpressing group and the control group have similar degrees of engraftment of *MLL-AF9* donor cells in BM at their end points, the former has a significantly lower population of the Mac-1^+^/Gr-1^+^ leukemic blast cells than the latter (Fig. 2g,h). Tissue staining showed that, compared with the control group, the BM cells in the *Alox5* overexpressing group were more differentiated, consistent with the flow cytometry results, and the infiltration of leukemic cells into spleen, liver, and peripheral blood was less severe (Fig. 2i). Thus, our data indicated that *Alox5* exhibited a moderate anti-tumor effect in the maintenance of *MLL*-rearranged AML and restrains leukemic cell infiltration.

### Forced expression of *Alox5* sensitizes *MLL*-rearranged AML cells to standard chemotherapy

**Figure 3:**
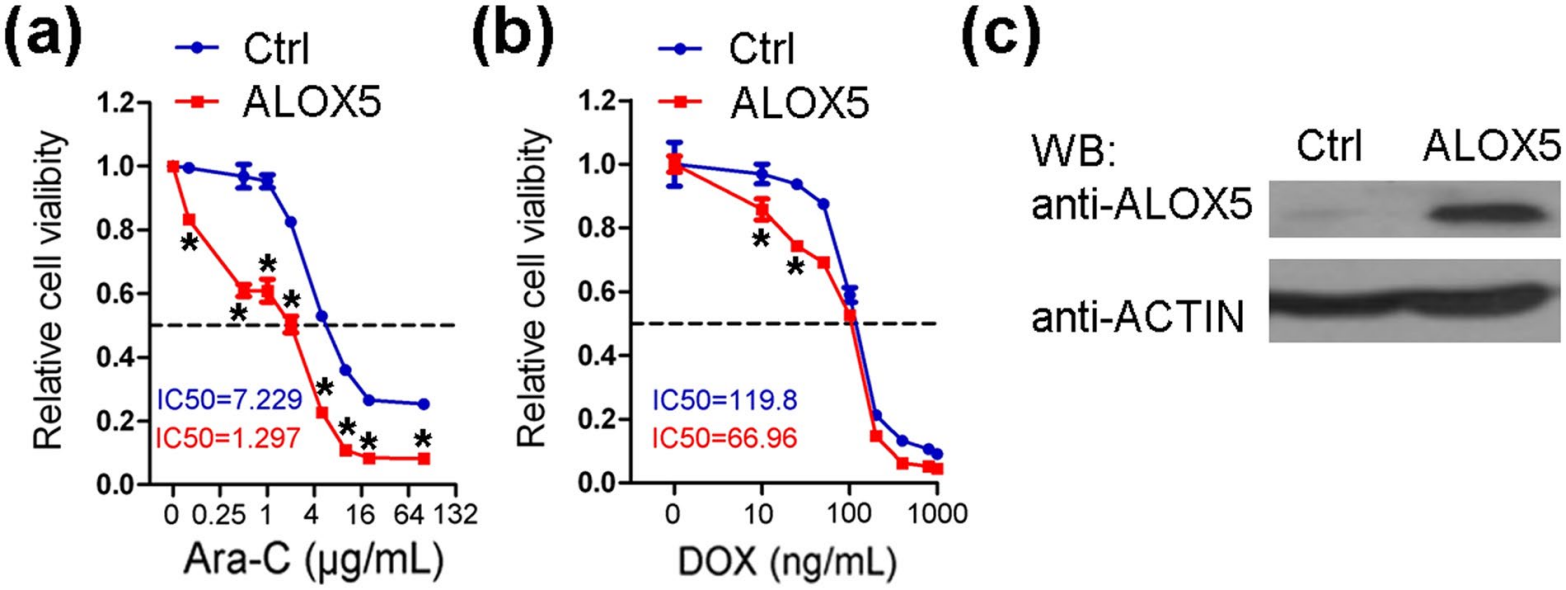
Drug sensitizing effect of *ALOX5* in human AML cells. (**a**,**b**) MONOMAC-6 cells were transduced with *ALOX5* or control lentivirus, and were treated with Ara-C (**a**) or DOX (**b**) at indicated doses 7 days after lentiviral infection and selection. MTT assays were conducted 72 hours post-drug treatments. *P < 0.05, two-tailed *t*-test. (**c**) Protein level of ALOX5 in the transduced cells was verified through Western blotting.

The anti-tumor role of *Alox5* implies a therapeutic potential in treating AML. Since restoration of *Alox5* expression/function alone showed only moderate inhibitory effect on AML progression, we sought to investigate whether *Alox5* restoration could facilitate chemotherapeutic response and thus yield a more effective therapeutic effect. We first used lentivirus to overexpress human *ALOX5* in MONOMAC-6/t(9;11) AML cells, and treated the transduced cells with doxorubicin (DOX) or cytarabine (Ara-C). Our results showed that overexpression of *ALOX5* significantly sensitized MONOMAC-6 cells to DOX and especially Ara-C treatment (Fig. 3a–c; Supplementary Fig. 3c,d).

**Figure 4:**
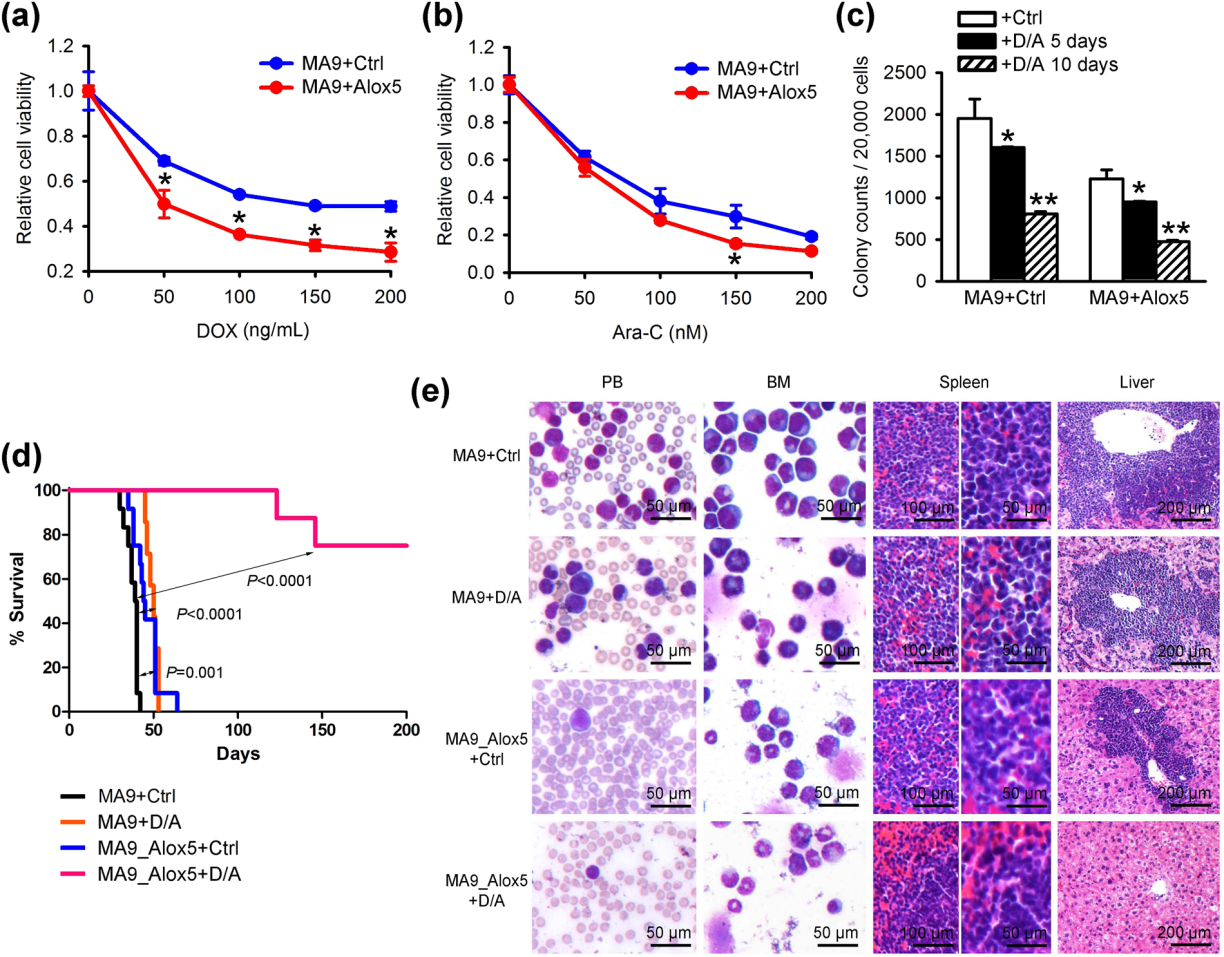
Drug sensitizing effect of *Alox5* in mouse *MLL*-rearranged AML. (**a**,**b**) Normal mouse bone marrow progenitor cells were retrovirally transduced with MSCVneo-*MLL-AF9* + MSCV-PIG (MA9 + Ctrl) or MSCVneo-*MLL-AF9* + MSCV-PIG-*Alox5* (MA9 + Alox5). 10,000 of the transduced cells were seeded in liquid culture medium and treated with DOX (**a**) or Ara-C (**b**) at indicated doses. MTT assays were conducted 72 hours after drug treatments. (**c**) *Alox5* overexpression enhanced the inhibitory effect of DOX and Ara-C on *MLL-AF9*-induced colony forming. On the second passage of serial replating, colony cells were treated with control, or 50 ng/mL DOX plus 50 nM Ara-C, for 5 or 10 days. *P < 0.05; **P < 0.01, two-tailed *t*-test. (**d**) *Alox5* overexpression enhanced the therapeutic effects of DOX and Ara-C on *MLL-AF9*-AML *in vivo*. Secondary *MLL-AF9*-AML recipient mice with *Alox5* overexpression (MA9_Alox5) or without (MA9) were treated with PBS (control), or a daily dose of 50 mg/kg Ara-C for five days along with a daily dose of 1.5 mg/kg DOX during the first three days of Ara-C treatment (i.e., ‘5 + 3’ regimen). Kaplan-Meier curves are shown. MA9 + Ctrl: n = 12; MA9 + DOX/Ara-C (D/A): n = 7; MA7_Alox5 + Ctrl: n = 12; MA9_Alox5 + D/A: n = 8. The *P* values were determined by log-rank test. (**e**) Wright-Giemsa staining of mouse PB and BM, or H&E staining of mouse spleen and liver of secondary BMT recipients after control or drug treatments.

Similarly, in mouse BM progenitor cells transduced with *MLL-AF9*, co-expression of *Alox5* remarkably enhanced the inhibitory effect of DOX or Ara-C on cell viability and colony forming capacity (Fig. 4a–c). We further tested the drug sensitizing effect of *Alox5 in vivo*. We transplanted *MLL-AF9* AML cells with forced expression of *Alox5* (i.e., MA9_Alox5) or without (i.e., MA9) into secondary recipient mice, and after the onset of leukemia, the recipient mice were treated with or without DOX + Ara-C (i.e. “5 + 3” regimen^5^). As shown in Fig. 4d, while DOX + Ara-C treatment alone only moderately (though statistically significantly) improved survival in mice carrying *MLL-AF9* AML, forced expression of *Alox5* dramatically improved the response of *MLL-AF9* AML to the DOX + Ara-C treatment (Fig. 4d). As a result, amazingly over 70% of mice in the MA9_Alox5 + DOX/Ara-C group survived over 200 days, while all mice in the other groups died from AML within 70 days (Fig. 4d). The infiltration of leukemic cells into the spleen, liver, and peripheral blood was almost completely blocked in the MA9_Alox5 + DOX/Ara-C group of mice (Fig. 4e). Therefore, restoration of *Alox5* holds great potential in improving chemotherapeutic response in *MLL*-rearranged AML patients.

### Signaling pathways correlated with *Alox5* overexpression in AML

**Figure 5:**
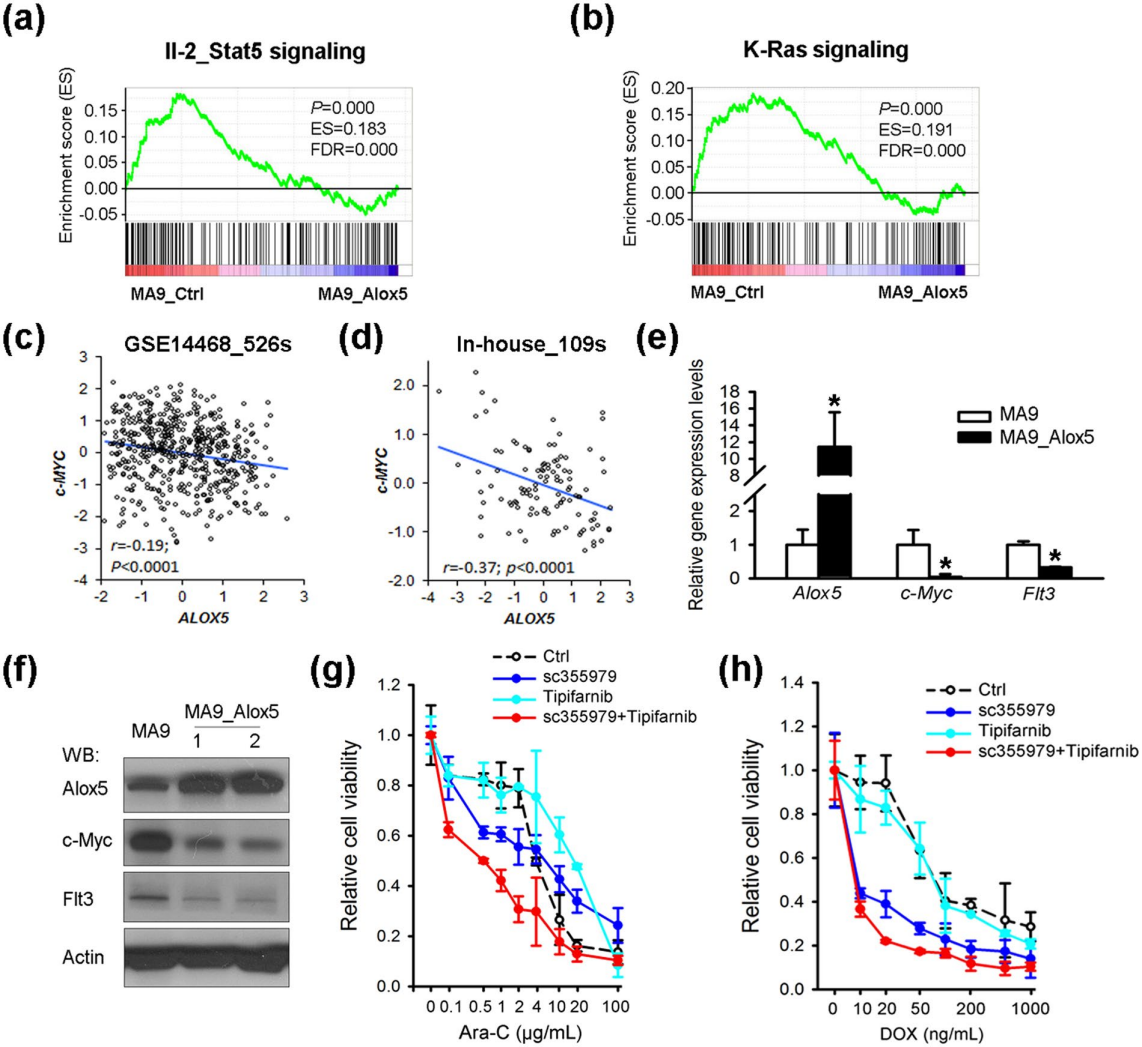
ALOX5 represses both STAT5 and K-RAS signaling pathways in *MLL*-rearranged AML. (**a**,**b**) RNA sequencing and Gene Set Enrichment Analysis (GSEA) of *Alox5* overexpressed AML. Shown are gene sets of Il-2/Stat5 pathway (**a**) and K-Ras pathway (**b**) that are significantly enriched (*P* < 0.05) in genes with a lower level in the BM leukemic blast cell samples of secondary *MLL-AF9*-AML mice with *Alox5* overexpression (MA9_Alox5), as compared with secondary *MLL-AF9*-AML mice without *Alox5* overexpression (MA9_Ctrl). ES, enrichment score; FDR, false discovery rate. (**c**,**d**) Correlation between the expression levels of *ALOX5* and *c-MYC*, a downstream target genes of shared by STAT and K-RAS signaling pathways, in two independent AML patient datasets. All expression data were log(2) transformed. The correlation coefficient (*r*) and *P* values were detected by “Pearson Correlation”, and the correlation regression lines were drawn with the “linear regression” algorithm. (**e**,**f**) Levels of Alox5, Myc and Flt3 in BM mononuclear cells of *MLL-AF9* leukemic mice and normal controls were detected with qPCR (**e**) and Western blotting (**f**). (**g**,**h**) Inhibition of STAT5 and K-RAS signaling pathways sensitizes MONOMAC-6 cells to chemotherapy. MONOMAC-6 cells were treated with DMSO control, 1 μM sc-355979, 100 nM Tipifarnib, in combination with control, Ara-C (**g**) or DOX (**h**) at indicated doses. Cell viability was tested 48 hours post treatment.

To delineate the potential molecular mechanism underlying the anti-tumor and drug-sensitizing effects of Alox5, we performed RNA sequencing (RNA-seq) of two pairs of mouse BM leukemic blast cells collected from the MA9_Ctrl and MA9_Alox5 mice in secondary BMT assays shown in Fig. 2d. Through gene set enrichment analysis (GSEA)^36^, we found that Il-2/Stat5 signaling and K-Ras pathway were significantly suppressed in MA9_Alox5 AML cells as compared with MA9_Ctrl AML cells (Fig. 5a,b). *Stat5* and *K-Ras* function as critical oncogenes in both solid tumors and leukemia, and both STAT5 and K-RAS signaling pathways were known to be closely associated with oncogenesis and drug sensitivity^15, 16, 37^. *c-MYC* is a downstream target gene shared by STAT and K-RAS signaling pathways^38, 39^. We analyzed two independent AML patient databases and found a significant negative correlation between the expression levels of *ALOX5* and *c-MYC* (Fig. 5c,d). In BM leukemic blast cells of *MLL-AF9* leukemic mice, overexpression of *Alox5* suppressed the expression of c-Myc and Flt3, an upstream key regulator of both Stat5 and K-Ras signaling^40^ (Fig. 5e,f; Supplementary Fig. 3e–g). In order to determine whether repression of STAT5 and/or K-RAS pathways could mimic the drug sensitizing effect of ALOX5, we treated MONOMAC-6 cells with a combination of DOX or Ara-C, together with control, STAT5 inhibitor sc-355979^41^, and/or RAS signaling inhibitor Tipifarnib^42^. Results showed that a combined treatment of both sc-355979 and Tipifarnib significantly sensitized the cells to chemotherapy (Fig. 5g,h). In a data set composed of 82 AML samples (including 26 *MLL*-rearranged AMLs)^43^, those bearing *K-RAS* mutations (n = 5) are associated with a lower *ALOX5* level (Supplementary Fig. 2a), indicating a potential positive feedback between K-RAS signaling and *ALOX5*. Noticeably, the only AML sample with mutated *K-RAS* that has relatively higher *ALOX5* level also bears *FLT3* mutation. The remarkable suppression of *ALOX5* in *MLL-*rearranged AML bearing *K-RAS* mutation was further verified in human CD34^+^ derived *MLL-AF9* cell lines (Supplementary Fig. 2b). Therefore, our results suggest that Alox5 exerts its anti-tumor and drug sensitizing effects in *MLL*-rearranged AML through suppressing the Stat and K-Ras signaling pathways.

## Discussion

In contrast to the oncogenic role of *ALOX5* reported previously in CML^26^, here we show that *ALOX5*, suppressed by PRC2 at the transcription level, exhibits a moderate anti-tumor effect both *in vitro* and *in vivo* in *MLL*-rearranged AML, in which *ALOX5* expression is especially repressed. More interestingly, we show that restoration of *Alox5* expression can substantially increase the sensitivity of *MLL*-rearranged AML cells to standard chemotherapeutic agents such as DOX and Ara-C both *in vitro* and *in vivo*, as well as the underlying mechanism through suppressing the STAT and K-RAS oncogenic signaling pathways.

Interestingly, *ALOX5* was largely known as an oncogene in solid tumors, e.g. prostate cancer and pancreas cancer^24, 25^. However, its role remains vague in leukemia. It was reported that *Alox5* deficiency resulted in a significant reduction of LSCs in BM, and thus largely prolonged survival of *BCR-ABL*-induced CML mice^26^. Nonetheless, there was no significant influence of *Alox5* deficiency on normal hematopoietic stem cells (HSCs) or on the induction of lymphoid leukemia by *BCR-ABL* ^26^. Another study showed that *Alox5* deficiency inhibited *in vitro* colony-forming capacity of BM progenitor cells induced by some AML oncogenic fusion genes such as *AML1-ETO9a*, a potent oncogenic isoform of *AML1-ETO* resulting from t(8;21)^44^, but had no significant effects on *AML1-ETO9a*-induced leukemogenesis *in vivo* ^32^. Those findings together with our present results suggest that *Alox5* likely plays distinct roles at different lineages (myeloid and lymphoid) or different stages (i.e. induction and maintenance) of various hematopoietic malignancies.

One of the major challenges in AML therapy is the high frequency of occurrence of therapeutic resistance and the subsequent relapse^7^. Thus far, a variety of factors, such as JAK/STAT signaling and RAS pathway, have been identified to be closely related with chemotherapy response^9, 13, 16^. Abnormal activation of JAK/STAT signaling is found in most *de novo* AML^13^. *K-RAS* is a proto-oncogene essential for many solid tumors and hematopoietic malignancies^16^. Although *RAS* (i.e. *N-RAS* or *K-RAS*) gene mutations were only found in no more than 10% of AML cases, activation of RAS pathway by mutations in upstream receptors, e.g. *FLT3* and *c-KIT*, or downstream effectors, broadly exist in AML^45^. A set of inhibitors have been developed to target JAK/STAT signaling (e.g. OPB-31121 and pacritinib) or RAS pathway (e.g., Selumetinib), but their efficacy in clinical trials to treat hematopoietic malignancies was still limited^46–48^. Our data suggest that restoration of *ALOX5* expression/function could suppress both JAK/STAT and RAS signaling pathways simultaneously, and thus represents an alternative strategy, other than individual small-molecule inhibitors of JAK/STAT and RAS signaling pathways, to target these two critical oncogenic pathways in treating AML. Especially, restoration of *ALOX5* expression/function in combination with the standard chemotherapy represents a potentially more effective therapeutic strategy for curing AML, at least *MLL*-rearranged AML. Thus, it would be interesting to identify small-molecule compound(s), such as natural product(s), that can specifically induce endogenous expression of *ALOX5* expression in AML cells, and such compound(s) can be applied together with standard chemotherapy to treat *MLL*-rearranged AML.

## Methods

### Affymetrix exon array assays of human samples

As described previously^27–29^, a total of 100 human AML (including 30 t(8;21), 27 inv(16), 31 t(15;17) and 12 *MLL*-rearranged) and 9 normal BM samples (including 3 each of CD34^+^ hematopoietic stem/progenitor, CD33^+^ myeloid, and mononuclear cell (MNC) samples) were analyzed by use of Affymetrix GeneChip Human Exon 1.0 ST arrays (Affymetirx, Santa Clara, CA). Robust Multi-array Average (RMA)^49^ was used for the data normalization with Partek Genomics Suite (Partek Inc., St. Louis, MI). The complete microarray data set has been deposited into the GEO database under the accession code GSE34184 and GSE30285^43^.

### Plasmid construction

The CDS of *ALOX5*, encoding the human *ALOX5* gene, was amplified through PCR using primers 5′-ATAACCGGTCCACCATGGATTACAAGGATGACGATGACAAGCCCTCCTACACGGTC-3′ and 5′-AGCGAATTCTCAGATGGGCACACTGTTCGGA-3′, and then cloned into pLJM1-EGFP lentiviral vector (Addgene, Cambridge, MA). The CDS of mouse *Alox5* gene was amplified through PCR using primers 5′-AATCTCGAGCCACCATGGATTAC AAGGATGACGATGACAAGCCCTCCTACACG and 5′-ATTGAATTCTTAGATGGCTACGCTGTTGGGAAT-3, and then ligated into a retroviral vector, namely MSCV-PIG (i.e., MSCV-puro-IRES-GFP vector; bearing *GFP* gene), a kind gift from Drs. Gregory Hannon, Scott Hammond, and Lin He (Cold Spring Harbor Laboratory, Cold Spring Harbor, NY).

### Cell culture

MONOMAC-6 cells were maintained in RPMI 1640 supplemented with 10% FBS, 1% HEPES, 2 mM L-Glutamine, 100 × Non-Essential Amino Acid, 1 mM sodium pyruvate, 9 μg/ml insulin and 1% penicillin-streptomycin. Mouse leukemic cells were kept in RPMI 1640 supplemented with interleukin 3 (IL-3) and IL-6, 10 ng/ml; SCF 100 ng/ml; 10% FBS and 1% penicillin-streptomycin. siRNAs were transfected with Cell Line Nucleofector Kit V following program T-037, using the Amaxa Nucleofector Technology (Amaxa Biosystems, Berlin, Germany). Experiments were performed 48 h after transfection.

### Lentivirus production and infection

pMD2.G, pMDLg/pRRE, pRSV-Rev and pLJM1-EGFP plasmids were purchased from Addgene (Cambridge, MA). 0.5 μg pMD2.G, 0.3 μg pMDLg/pRRE, 0.7 μg pRSV-Rev and 1.5 μg pLJM1-EGFP constructs, i.e. pLJM1-*ALOX5* or pLJM1-EGFP control were co-transfected into HEK-293T cells in 60 mm cell culture dish with Effectene Transfection Reagent (QIAGEN, Valencia, CA). Lentiviral particles were harvested at 48 and 72 hours after transfection and concentrated with PEG-it^TM^ Virus Precipitation Solution (SBI). The lentivirus particles were directly added into leukemic cells and the cells were washed with PBS 24 hours after infection.

### Cell viability assays

Cells were seeded into 96-well plates at the concentration of 10,000 cells/ well in triplicates and MTT (Promega, Madison, WI) was used to assess cell proliferation and viability following the manufacturer’s instructions.

### Chromatin immunoprecipitation (ChIP)

ChIP assay was conducted as described previously^50^, with SABiosciences Corporation’s ChampionChIP One-Day kit (Qiagen, Frederick, MD) following the manufacturer’s protocol. Chromatin from THP-1 cells were cross-linked, sonicated into an average size of ~500 bp, and then immunoprecipitated with antibodies against the N’-terminal of MLL (MLL-N), the C’-terminal of MLL (MLL-C), EZH2, SIN3A, H3K27Me3 or IgG (Abcam, Cambridge, MA). Purified DNA was amplified by real-time qPCR using primers targeting the promoter of *ALOX5* as described before^32^.

### Western blotting

Cells were washed twice with ice-cold phosphate-buffered saline (PBS) and ruptured with RIPA buffer (Pierce, Rockford, IL) containing 5 mM EDTA, PMSF, cocktail inhibitor, and phosphatase inhibitor cocktail. Cell extracts were microcentrifuged for 20 min at 10,000 g and supernatants were collected. Cell lysates (20 μl) were resolved by SDS-PAGE and transferred onto PVDF membranes. Membranes were blocked for 1 hour with 5% skim milk in Tris-buffered saline containing 0.1% Tween 20 and incubated overnight at 4 °C with anti-ALOX5 antibody (Cell Signaling Technology Inc., Danvers, MA) or anti-ACTIN antibody (Santa Cruz Biotechnology Inc., Santa Cruz, CA). Membranes were washed 30 min with Tris-buffered saline containing 0.1% Tween-20, incubated for 1 hour with appropriate secondary antibodies conjugated to horseradish peroxidase, and developed using chemiluminescence substrates.

### *In vitro* colony forming and replating assays (CFAs)

CFAs were conducted as described previously with some modifications^29, 50, 51^. Briefly, retrovirus vectors were co-transfected with pCL-Eco packaging vector (IMGENEX, San Diego, CA) into HEK293T cells using Effectene Transfection Reagent (Qiagen, Valencia, CA) to produce retrovirus. BM cells were harvested from a cohort of 4- to 6-week-old B6.SJL (CD45.1) donor mice after five days of 5- fluorouracil (5-FU) treatment, and primitive hematopoietic progenitor cells were enriched with Mouse Lineage Cell Depletion Kit (Miltenyi Biotec Inc., Auburn, CA). An aliquot of enriched hematopoietic progenitor cells was added to retroviral supernatant together with polybrene in cell culture plates, which were centrifuged at 2,000 g for 2 hours at 32 °C (i.e., “spinoculation”^27–29^) and then the medium was replaced with fresh media and incubated for 20 hours at 37 °C. Next day, the same procedure was repeated once.

Then, on the day following the second spinoculation, an equivalent of 2.0 × 10^4^ cells were plated into a 35 mm Petri dish in 1.5 ml of Methocult M3230 methylcellulose medium (Stem Cell Technologies Inc, Vancouver, Canada) containing 10 ng/ml each of murine recombinant IL-3, IL-6, and granulocyte-macrophage colony-stimulating factor (GM-CSF), and 30 ng/ml of murine recombinant SCF (R&D Systems, Minneapolis, MN), along with 1.0 mg/ml of G418 and 2 μg/ml of puromycin. For each transduction, there were two duplicate dishes. Cultures were incubated at 37 °C in a humidified atmosphere of 5% CO_2_ in air. The colonies were replated every 7 days under the same conditions. The colony-forming/replating assays were repeated 3 times.

### The maintenance, monitoring, and end-point treatment of mice

C57BL/6 (CD45.2), B6.SJL (CD45.1) mice were purchased from the Jackson Lab (Bar Harbor, ME, USA) or Harlan Laboratories, Inc (Indianapolis, IN, USA) and maintained in house. Both male and female mice were used for the experiments. All laboratory mice were maintained in the animal facility at University of Cincinnati and the University of Chicago. All experiments on mice in our research protocol were approved by Institutional Animal Care and Use Committee (IACUC) of University of Cincinnati and the University of Chicago. All methods were performed in accordance with the relevant guidelines and regulations. The maintenance, monitoring, and end-point treatment of mice were conducted as described previously^27–29, 50, 52–54^.

### Bone marrow transplantation assays

For primary bone marrow transplantation (BMT) assays, normal BM cells of B6.SJL (CD45.1) mice were retrovirally co-transduced with MSCVneo-*MLL-AF9* + MSCV-PIG (i.e., MA9 + Ctrl) or MSCVneo-*MLL-AF9* + MSCV-PIG-*Alox5* (i.e., MA9 + Alox5), through two rounds of spinoculation, as described previously^27–29, 50, 52–54^. Then, the transduced donor cells were injected by tail vein into lethally irradiated (960 rads) 8- to 10-week-old C57BL/6 (CD45.2) recipient mice with 3 × 10^5^ donor cells plus a radioprotective dose of whole BM cells (1 × 10^6^; freshly harvested from a C57BL/6 mouse) per recipient mouse.

For secondary BMT assays, leukemic BM cells (with CD45.1^+^ percentage >90%) were isolated from primary leukemic mice (CD45.2) that received BMT of wild-type BM progenitor donor cells (CD45.1) infected with MSCVneo-*MLL-AF9* retrovirus alone. The leukemic cells from the primary recipients were retrovirally transduced with MSCV-PIG (MA9_Ctrl) or MSCV-PIG-*Alox5* (MA9_Alox5), and then transplanted into sublethally irradiated (480 rads) 8- to 10-week-old C57BL/6 (CD45.2) secondary recipient mice *via* tail vein injection. 1.5 × 10^5^ donor cells were transplanted into each secondary recipient mouse.

Peripheral blood (PB) cells were collected from the transplanted recipient mice monthly *via* tail bleeding and at any time that the mice showed signs of systemic illness to obtain the complete blood counts with white blood cell (WBC) differentials and the blood smears for the presence of immature or abnormal hematopoietic cells. The engraftment was assessed by flow cytometry analysis of CD45.1 in PB. Leukemic mice were euthanized by CO_2_ inhalation if they showed signs of systemic illness to collect specimens as controls for further analyses. The spleen, liver and thymus were weighed. Cells were obtained from PB, BM, spleen, and liver for flow cytometric analysis. Blood smear and BM cytospin slides were stained with Wright-Giemsa. For histological analysis, portions of the spleen and liver were fixed in formalin, sectioned, embedded in paraffin, and stained with hematoxylin and eosin (H&E).

### Gene set analyses

Gene Set Enrichment Analysis (GSEA)^36^ was used to analyze the signal pathway enrichment in different groups of samples. “Hallmark gene sets” obtained from MsigDB (The Molecular Signatures Database) were used as the “gene sets database” input. Vertebrate homology resource from the Mouse Genome Database (MGD)^55^ was extracted to convert between homologous human and mouse gene symbols.

### Statistical software

The gene/exon array data analyses and qPCR data analyses were conducted by use of Partek Genomics Suite (Partek Inc, St. Louis, MI), TIGR Mutiple Array Viewer software package (TMeV version 4.6; TIGR, Rockville, MA)^56^, and/or Bioconductor R packages. The t-test, Kaplan-Meier method, and log-rank test, etc. were performed with WinSTAT (R. Fitch Software), GraphPad Prism version 5.0 (GraphPad Software, San Diego, CA), and/or Partek Genomics Suite (Partek Inc, St. Louis, MI). The *P*-values less than 0.05 were considered as statistically significant. Significance analysis of microarrays (SAM)^57^, embedded in the TMeV package (TIGR, Rockville, MA), was used to identify the genes that are significantly (*q* < 0.05; false discovery rate, FDR < 0.05) dysregulated in human AML samples relative to the normal controls.

### Data availability

Data referenced in this study are available in The Gene Expression Omnibus. The Affymetrix exon array data and the microarray data are available under accession codes code GSE34184 and GSE30285. The mouse RNA sequencing data is available under accession code GSE94840. The RNA sequencing results of the AML sample cohort analyzed for the correlation of *ALOX5* levels and *K-RAS* mutations is under accession code GSE62190.

## Electronic supplementary material

Supplementary Information
