# Supplementary material for: ALOX5 exhibits anti-tumor and drug-sensitizing effects in MLL-rearranged leukemia
Source: Sci Rep. 2017 May 12;7:1853. doi: 10.1038/s41598-017-01913-y (PMC5431828; doi:10.1038/s41598-017-01913-y)
Supplement: Supplementary file 1 — Supplementary Information [file 41598_2017_1913_MOESM1_ESM.pdf]

Supplementary Information

ALOX5 exhibits anti-tumor and drug-sensitizing effects in *MLL*-rearranged leukemia

Yungui Wang, Jennifer R. Skibbe, Chao Hu, Lei Dong, Kyle Ferchen, Rui Su, Chenying Li, Hao Huang, Hengyou Weng, Huilin Huang, Xi Qin, Jie Jin, Jianjun Chen, Xi Jiang

<u>Inventory of Supplementary Information</u>

Supplementary Figures 1-3 and Figure Legends (Page 2-4)

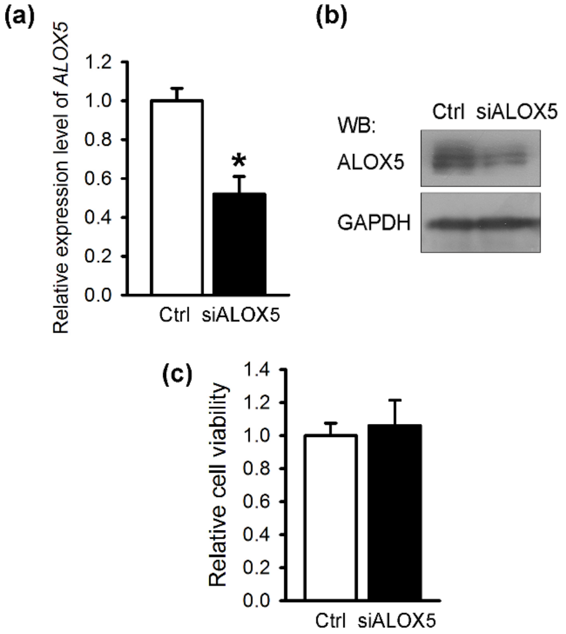

Supplemental Figure 1. Knockdown of *ALOX5* shows no significant effect on AML cell viability. (**a**) MONOMAC-6 cells were transfected with control siRNA or *ALOX5* siRNA. qPCR was performed to detect the expression level of *ALOX5* 48 hrs post transfection. (**b**) Inhibitory effect of *ALOX5* siRNA in MONOMAC-6 cells on ALOX5 protein level. (**c**) Effects of *ALOX5* siRNA on the viability of MONOMAC-6 cells. Cell viability was tested 48 hrs post transfection. *P<0.05, two-tailed *t*-test.

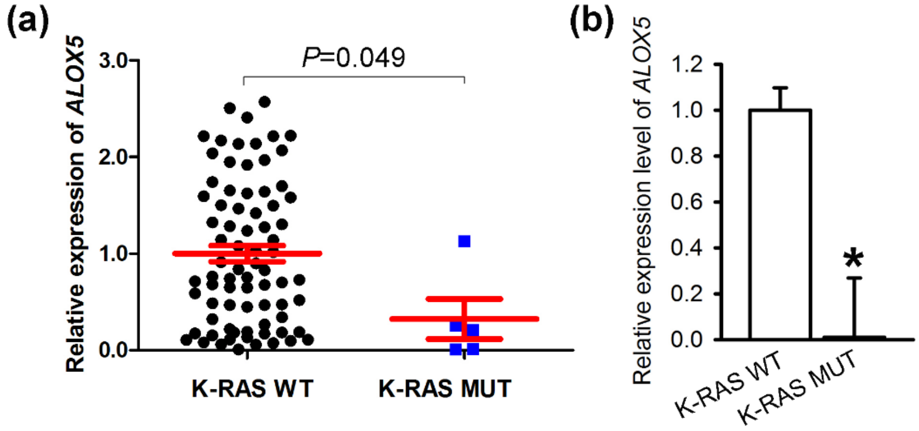

Supplemental Figure 2. *K-RAS* mutations repress *ALOX5* level in AML. (**a**) Comparison of *ALOX5* expression between AML with wild-type K-RAS and K-RAS mutations (K-RAS MUT) in GSE62190. (**b**) Expression levels of *ALOX5* in human $CD34^+$-derived *MLL-AF9* cell lines with or without *K-RAS* mutation. *$P<0.05$, two-tailed *t*-test.

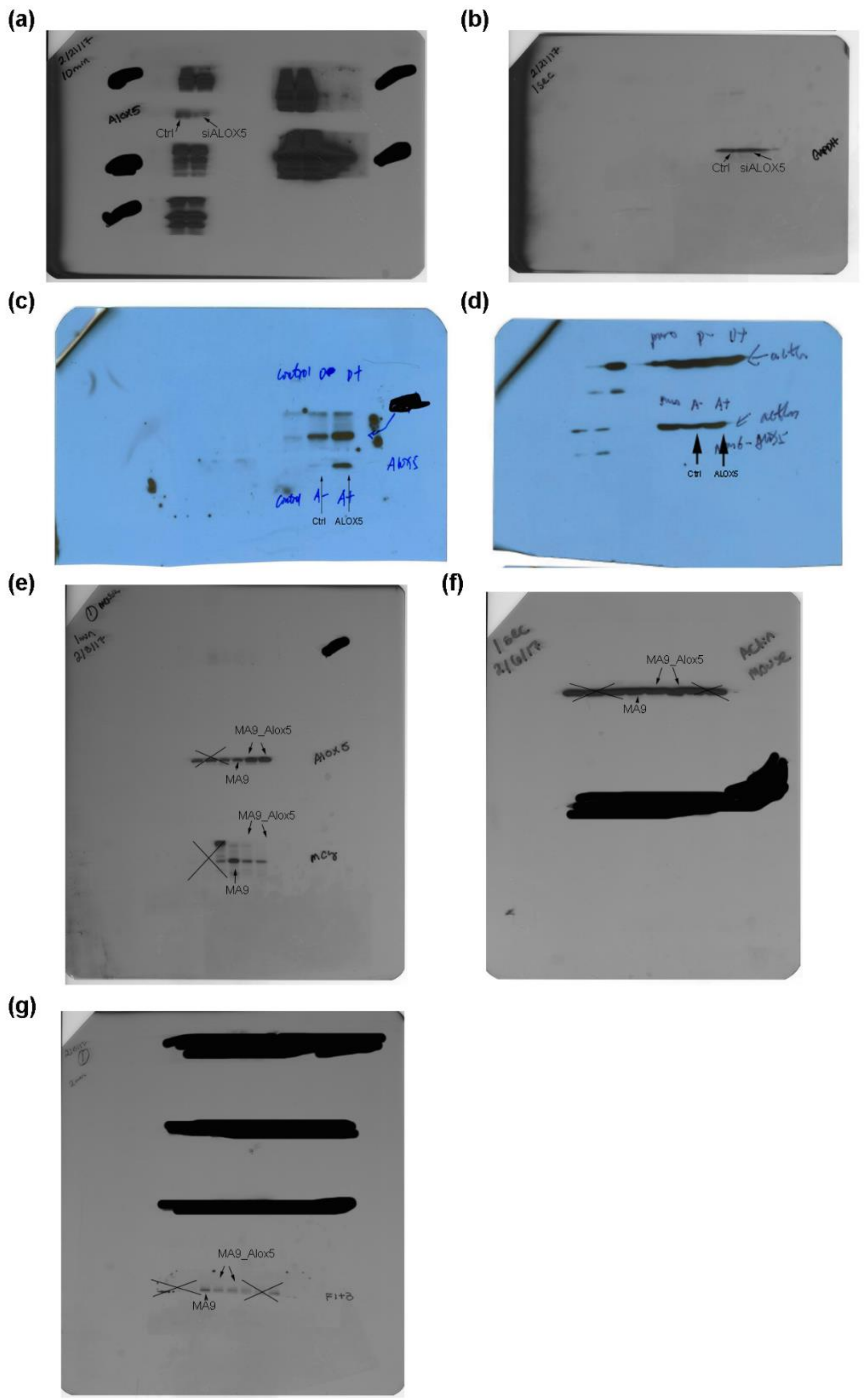

Supplementary Figure 3. Original scans of Western blotting results.
